# Antenatal corticosteroid treatment for women with hypertensive disorders of pregnancy: A population‐based study in Japan

**DOI:** 10.1111/jog.16364

**Published:** 2025-07-02

**Authors:** Takafumi Ushida, Kazuya Fuma, Satoru Katsuki, Sho Tano, Seiko Matsuo, Kenji Imai, Hiroaki Kajiyama, Tomomi Kotani

**Affiliations:** ^1^ Department of Obstetrics and Gynecology Nagoya University Graduate School of Medicine Nagoya Japan; ^2^ Division of Reproduction and Perinatology Center for Maternal‐Neonatal Care, Nagoya University Hospital Nagoya Japan; ^3^ Department of Obstetrics and Gynecology Japanese Red Cross Aichi Medical Center Nagoya Daini Hospital Nagoya Japan; ^4^ Department of Obstetrics and Gynecology Hamamatsu University School of Medicine Hamamatsu Japan

**Keywords:** antenatal corticosteroid, hypertensive disorders of pregnancy, preeclampsia

## Abstract

**Aim:**

This study aimed to evaluate the current antenatal corticosteroid (ACS) treatment practices for hypertensive disorders of pregnancy (HDP) in Japan, by evaluating annual trends and identifying clinical disparities and factors influencing non‐administration of ACS.

**Methods:**

This retrospective population‐based study was conducted using the Japanese Perinatal Research Network Database from 2013 to 2022. We analyzed ACS administration rates over time, across facility types, and the timing of delivery. Factors influencing non‐administration were identified using univariate and multivariate logistic regression analyses.

**Results:**

ACS administration rates among patients with HDP nearly doubled over the past decade, reaching 64.0% in 2022. Approximately 70% of patients with HDP who received ACS delivered before 34 weeks of gestation; however, only 30% achieved the optimal administration‐to‐birth interval of 48 h to 7 days. ACS administration rates in patients with HDP differed by facility type: 67.8% (1641/2419) in general perinatal medical centers, 60.5% (1107/1830) in regional perinatal medical centers, and 39.7% (23/58) in non‐perinatal medical centers. Factors contributing to non‐administration included smoking during pregnancy, eclampsia, placental abruption, HELLP syndrome, regional perinatal medical centers, non‐perinatal medical centers, and gestational hypertension. Conversely, conditions such as fetal growth restriction, threatened preterm labor, and preterm premature rupture of membranes were associated with higher rates of ACS administration.

**Conclusions:**

Although ACS administration rates in patients with HDP have improved over time, challenges remain in achieving optimal administration timing and addressing facility‐based disparities. To improve administration rates, clinicians should increase awareness of ACS treatment and proactively manage HDP‐related emergencies.

## INTRODUCTION

Hypertensive disorders of pregnancy (HDP) represent a spectrum of conditions characterized by elevated blood pressure during pregnancy and are classified as gestational hypertension, preeclampsia, chronic hypertension, and superimposed preeclampsia (SPE).^1^, ^2^ The etiology of HDP is multifactorial, involving abnormal placentation, immune maladaptation, and systemic endothelial dysfunction.^1^ These disorders, particularly preeclampsia, are associated with proteinuria, end‐organ dysfunction, and uteroplacental dysfunction.^3^, ^4^ HDP affect approximately 5%–10% of pregnancies worldwide and remain a leading cause of maternal and neonatal morbidity and mortality.^1^ Maternal complications include stroke, renal failure, liver dysfunction, and peripartum cardiomyopathy. Neonatal risks include preterm birth, small for gestational age, and preterm birth‐associated complications, such as neonatal mortality, respiratory distress syndrome (RDS), intraventricular hemorrhage (IVH), and periventricular leukomalacia (PVL).^5^, ^6^

Antenatal corticosteroid (ACS) treatment is well‐established as an effective intervention for improving neonatal outcomes in pregnancies at risk of preterm delivery, typically before 34 weeks of gestation.^7^ ACS treatment primarily accelerates fetal lung maturation and significantly reduces the risk of RDS in preterm infants. Additionally, ACS treatment reduces the incidence of various neonatal complications, including neonatal mortality, IVH, and PVL, by approximately 20%–50%.^7^, ^8^ Therefore, ACS treatment remains a cornerstone in the management of threatened preterm labor, HDP, and fetal growth restriction, with its benefits firmly established through robust clinical data.^7^ Leading organizations, including the World Health Organization and the American College of Obstetricians and Gynecologists, strongly recommend ACS treatment.^9^, ^10^ In Japan, ACS treatment was approved under insurance coverage in 2009.

Regarding HDP management, timely diagnosis, antihypertensive therapy, fetal monitoring, and appropriate delivery planning for emergency complications are crucial for mitigating both maternal and fetal complications.^2^ However, except for iatrogenic delivery, there is currently no definitive or effective treatment for HDP. Furthermore, when iatrogenic preterm delivery is required owing to maternal or neonatal indications, the risk of various neonatal complications associated with preterm birth increases, highlighting the importance of ACS treatment. We previously demonstrated that ACS treatment significantly decreased short‐term adverse outcomes (e.g., neonatal death, IVH, and PVL) in neonates born to mothers with HDP, with effectiveness comparable to that observed in neonates born to mothers without HDP.^11^

Despite the established benefits of ACS treatment, annual trends and inter‐facility variations in its administration rate for women with HDP in Japan, as well as the timing of delivery in ACS‐treated cases, remain unclear. Studying ACS treatment in patients with HDP may help identify patterns and barriers in clinical practice, thereby contributing to improved perinatal management. Moreover, limited information is available regarding the characteristics of patients with HDP who do not receive ACS treatment. Addressing these knowledge gaps may facilitate the development of targeted interventions to increase ACS administration rates and improve neonatal clinical outcomes in patients with HDP.

To address these gaps, this study aimed to investigate current practices of ACS treatment in patients with HDP using a population‐based perinatal database from the Japan Society of Obstetrics and Gynecology (JSOG). By analyzing this comprehensive dataset, we aimed to clarify current patterns in ACS treatment, identify potential disparities in its administration, and explore factors associated with non‐administration. Ultimately, our goal was to contribute to optimizing perinatal management strategies involving ACS treatment in patients with HDP, ensuring that opportunities to improve neonatal outcomes are not overlooked.

## MATERIALS AND METHODS

### Study population and data source

This retrospective population‐based study was conducted using the Perinatal Research Network Database established by the JSOG, which includes detailed perinatal information across obstetric institutions in Japan. This database was developed to collect and analyze perinatal clinical data to improve the quality of perinatal care, support medical research, and inform evidence‐based practices in maternal and neonatal health. The database includes data from approximately 400 participating facilities throughout Japan, covering 200 000–250 000 deliveries from 22 weeks onward, representing approximately 25% of all deliveries in Japan. The participating institutions comprised 95 of 110 general perinatal medical centers, 207 of 298 regional perinatal medical centers, and 106 non‐perinatal medical centers in 2019. Therefore, this database was primarily composed of data from high‐risk deliveries. The registry of this database was approved by the institutional ethics committee of each participating facility. Informed consent was not mandatory and was waived at most facilities due to the anonymization of all clinical data. The database includes detailed clinical data on more than 300 maternal, fetal, and neonatal characteristics such as maternal medical history, pregnancy complications, delivery outcomes, and neonatal outcomes. Information on medical interventions, such as ACS treatment, tocolytic agents, antihypertensive medications, and treatments for pregnancy‐related disorders are also included in the database. Since 2020, the perinatal registration system has been fully digitized, and the registry items have also been revised.

Clinical data between 2013 and 2022 were obtained from the Perinatal Research Network Database. Exclusion criteria included incomplete maternal medical records (e.g., gestational week at delivery, HDP, and ACS treatment) and stillbirths. Because this database is structured based on the data for each neonate, duplicate entries resulting from multiple pregnancies were excluded. Data entry for ACS treatment was recorded only when the ACS treatment was administered. Therefore, when ACS treatment is marked as “no,” it is impossible to distinguish between cases where ACS treatment was genuinely not administered and cases where ACS treatment was given but not recorded. Supplementary Figure 1 shows the number of facilities with 10 or more preterm deliveries before 34 weeks of gestation in 2022, categorized according to ACS administration rate for preterm deliveries. The ACS administration rates did not follow a normal distribution, with approximately 7.5% of the facilities reporting a rate of zero. To address this issue, we calculated the Z‐score (= [value – mean]/standard deviation) in this study and defined outliers as values with a *Z*‐score exceeding ±2. The red areas in this figure correspond to *Z*‐scores exceeding ±2, and these facilities were excluded. Clinical data from facilities identified as outliers in each year were excluded from the analysis. Use of this database was approved by the Executive Committee of JSOG (approval number: 169), and this retrospective study was approved by the Ethics Committee of Nagoya University Hospital (approval no. 2024‐301; approval date: November 11, 2024).

### Definition

HDP were defined as a systolic blood pressure of 140 mmHg or higher, or a diastolic blood pressure of 90 mmHg or higher during pregnancy.^12^, ^13^ This definition of HDP in Japan aligns with the international standards.^2^

### ACS treatment

Obstetric practice guidelines in Japan recommend ACS treatment consisting of two intramuscular injections of 12 mg betamethasone administered 24 h apart.^14^ The Perinatal Research Network Database provides detailed information regarding the dose (partial or full), type of corticosteroid (betamethasone or dexamethasone), and the interval between the first administration of ACS and delivery (< 48 h with a partial dose of ACS, <48 h with a full dose of ACS, 48 h to 7 days, 7 days to 1 month, more than 1 month, and unknown). Among women with an administration‐to‐birth interval of less than 48 h, a partial dose of ACS was defined as the administration of less than 24 mg of either betamethasone or dexamethasone, and a full dose of ACS was defined as the administration of 24 mg of either betamethasone or dexamethasone.

### Data analysis

**FIGURE 1:**
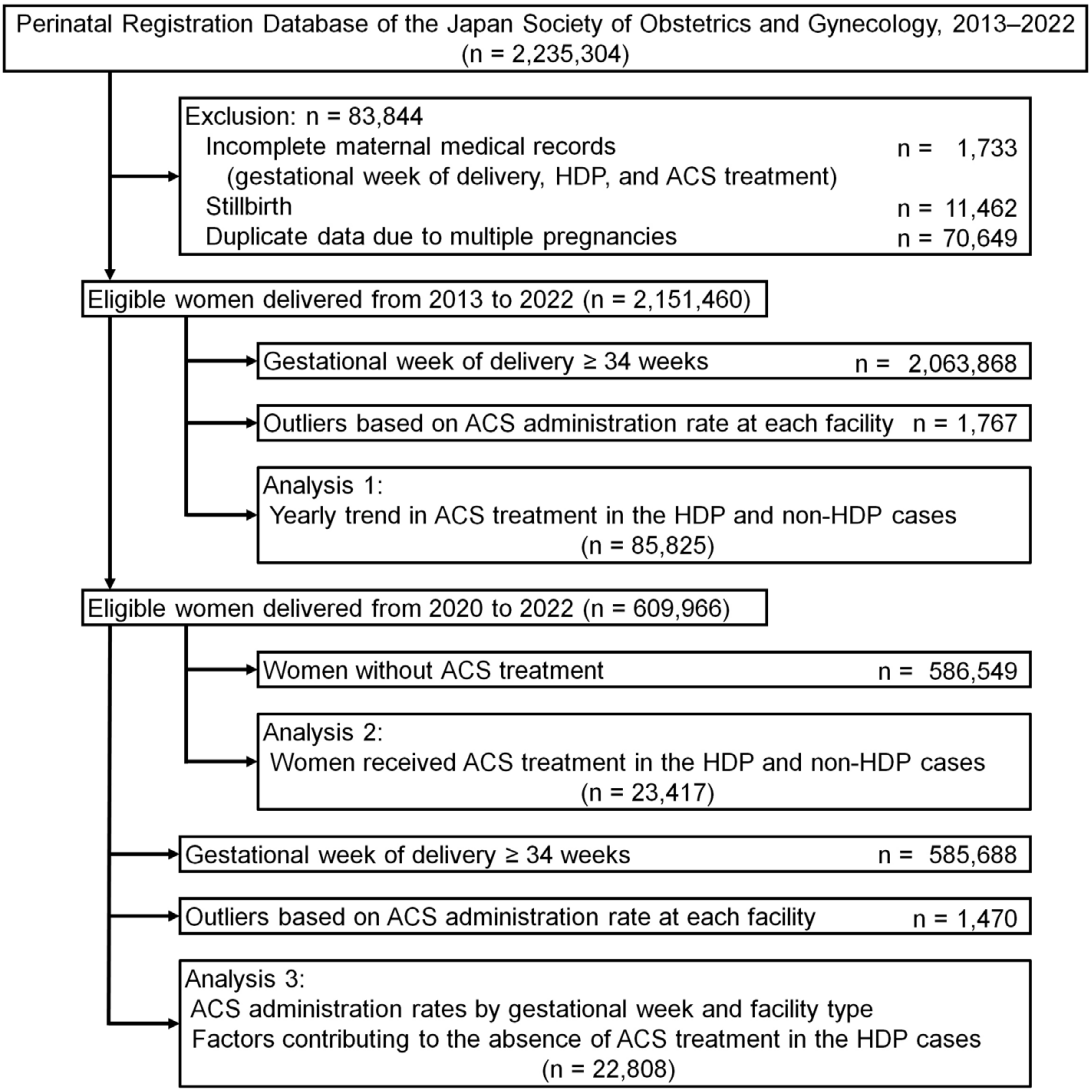
Flow diagram of study analysis. Clinical data of 2 235 304 women who delivered between 2013 and 2022 were collected, and 2 151 460 women were eligible for this study after excluding 83 844 women. Analysis 1 was conducted in women who delivered before 34 weeks of gestation to investigate the yearly trends in ACS treatment in HDP and non‐HDP cases. Analysis 2 was conducted for women who received ACS treatment to analyze the timing of delivery and administration‐to‐birth interval in the HDP and non‐HDP cases. Analysis 3 was conducted for women who delivered before 34 weeks of gestation from 2020 to 2022 to analyze the ACS administration rate according to the gestational week, facility type, and clinical factors influencing non‐ACS administration. ACS, antenatal corticosteroid; HDP, hypertensive disorders of pregnancy.

Three main analyses were performed (Figure 1). In Analysis 1, the annual ACS administration rates from 2013 to 2022 were evaluated in women who delivered before 34 weeks of gestation, stratified by the presence or absence of HDP. Both singleton and multiple pregnancies were included, and additional analyses were conducted for both situations. Additionally, the ACS administration rates among women who delivered before 28 weeks of gestation were assessed. Due to revisions made to the perinatal registration system in 2020, including changes in registered data items, subsequent analyses were conducted using the database from 2020 to 2022. In Analysis 2, clinical data from the perinatal database between 2020 and 2022 were used, focusing on women who underwent ACS treatment. Among these cases, the proportions of women who delivered before 34 weeks, between 34 and 36 weeks, and at 37 weeks or later were investigated. Additionally, the dose of ACS treatment and administration‐to‐birth interval were evaluated. In Analysis 3, clinical data from the perinatal database from 2020 to 2022 were used with a focus on women who delivered before 34 weeks of gestation. The ACS administration rates were evaluated across gestational weeks and facility types in patients with HDP. Univariate and multivariate logistic regression analyses were conducted to identify the factors associated with non‐administration of ACS in patients with HDP. Variables with a *p*‐value <0.10 in the univariate analysis were included in the multivariable analysis, and significant factors were identified using a forced entry method. Odds ratios (ORs) were evaluated using 95% confidence intervals (CIs). Feature importance was further assessed using the mean decrease in accuracy (MDA) with a machine learning method, the random forest algorithm. The MDA represents the decrease in model accuracy when a specific feature is permuted, reflecting the contribution of the feature to the prediction. The MDA was calculated 100 times, and the average values were used for the analysis. The features at the top of the ranking indicated a greater importance in predicting non‐ACS treatment. All statistical analyses were performed using the SPSS (version 29; SPSS Inc., Chicago, IL, USA) and R software (version 4.4.2; R Foundation for Statistical Computing).

## RESULTS

**FIGURE 2:**
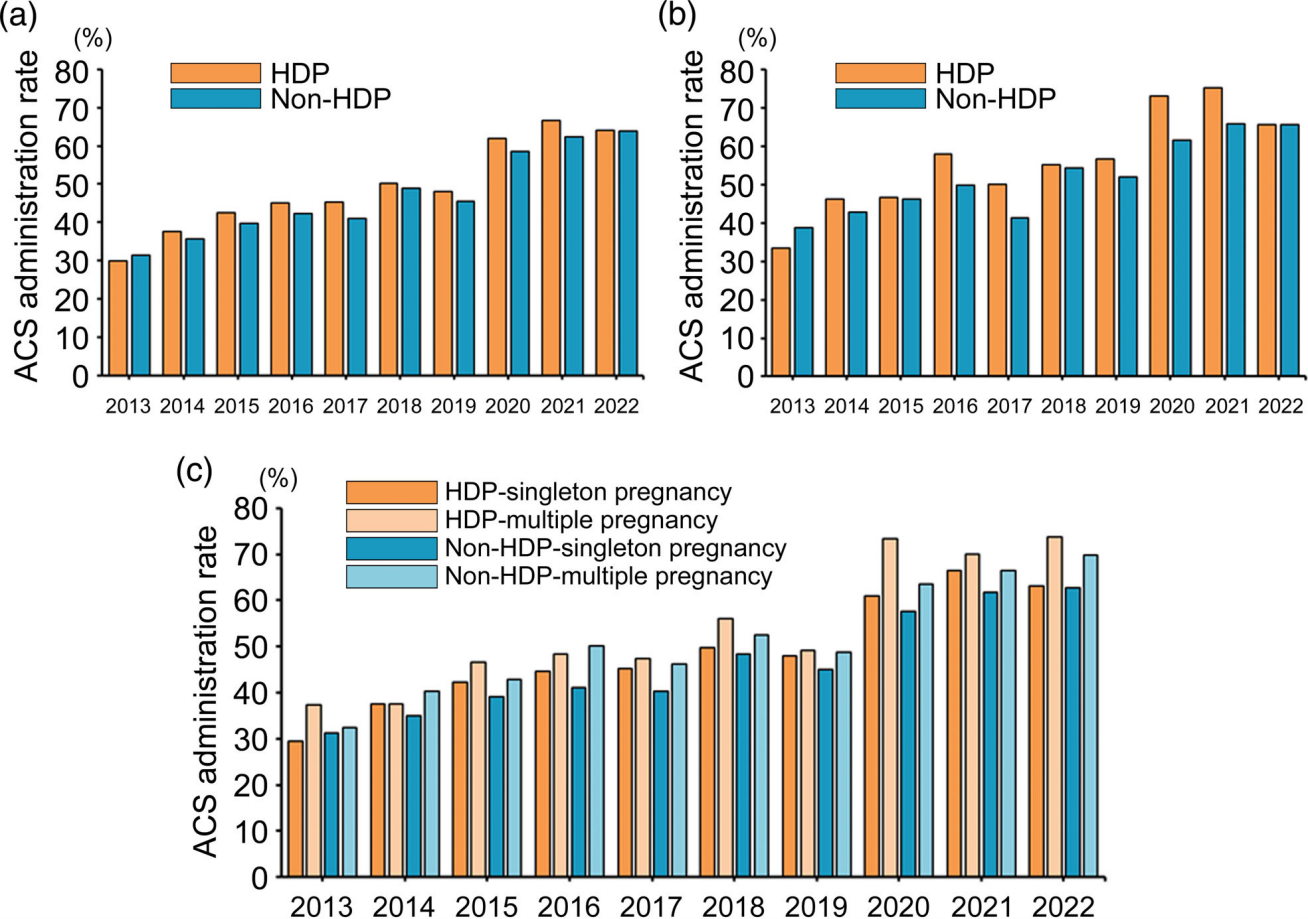
Annual trend of ACS administration rate in HDP and non‐HDP cases between 2013 and 2022 in Japan. (a) Annual trend of ACS administration rate in women delivered at less than 34 weeks of gestation between 2013 and 2022. (b) Annual trend of the ACS administration rate for women delivered at less than 28 weeks of gestation between 2013 and 2022. (c) Annual trend of the ACS administration rates for women delivered at less than 34 weeks of gestation in singleton and multiple pregnancies between 2013 and 2022. ACS, antenatal corticosteroid; HDP, hypertensive disorder of pregnancy.

Clinical data of 2 235 304 women who delivered children between 2013 and 2022 were collected; 2 151 460 women were eligible for this study after excluding 83 844 women. A flow diagram detailing participant selection and the number of eligible women included in Analyses 1–3 is shown in Figure 1. In Analysis 1, ACS administration rates for HDP and non‐HDP patients showed a steady increase from 2013 to 2022 (Figure 2a). In 2013, the ACS administration rate was 30.0% in HDP cases and 31.5% in non‐HDP cases; however, by 2022, these rates had nearly doubled to 64.0% and 63.8%, respectively. Overall, the ACS administration rate tended to be higher in patients with HDP; however, the difference was relatively small. Furthermore, the ACS administration rate in patients who delivered before 28 weeks of gestation (Figure 2b) was generally higher than those who delivered before 34 weeks of gestation (Figure 2a). In patients with HDP who delivered before 28 weeks of gestation, the ACS administration rate increased from 33.5% in 2013 to a peak of 75.2% in 2021. Similarly, in non‐HDP cases, the rate increased from 38.8% in 2013 to 65.8% in 2021. Figure 2c displays the ACS administration rates stratified by singleton and multiple pregnancies in both HDP and non‐HDP cases, showing a tendency toward higher ACS administration rates in multiple pregnancies compared to singleton pregnancies.

In Analysis 2, gestational weeks at delivery were evaluated in 23 417 patients who underwent ACS between 2020 and 2022, including 3894 patients with HDP and 19 523 without HDP (Table 1). Among the patients with HDP, 2771 (71.2%) delivered before 34 weeks and 264 (6.8%) delivered at 37 weeks or later. In contrast, for non‐HDP cases, 14 169 (58.4%) were delivered before 34 weeks and 3366 (15.9%) were delivered at 37 weeks or later. The proportion of deliveries before 34 weeks was significantly higher in the HDP group than that in the non‐HDP group. The administration‐to‐birth interval and dose of ACS treatment (< 48 h with a partial dose of ACS, <48 h with a full dose of ACS, 48 h to 7 days, 7 days to 1 month, >1 month, and unknown) were evaluated in patients with and without HDP (Table 2). Among patients with HDP, 31.9% were delivered between 48 h and 7 days after ACS administration, the period during which the therapeutic effect of ACS was the most pronounced. The optimal timing of delivery was significantly higher in patients with HDP than in those without HDP.

**TABLE 1:** Distribution of gestational weeks at delivery in HDP and non‐HDP cases with ACS treatment in 2020–2022.

	< 34 weeks	34–36 weeks	≥ 37 weeks	Total
HDP	2771 (71.2)	859 (22.1)	264 (6.8)	3894 (100)
Non‐HDP	11 398 (58.4)	5023 (25.7)	3102 (15.9)	19 523 (100)
Total	14 169 (60.5)	5882 (25.1)	3366 (14.4)	23 417 (100)

*Note*: Data are presented as *n* (%).

Abbreviations: ACS, antenatal corticosteroid; HDP, hypertensive disorders of pregnancy.

**TABLE 2:** Timing of delivery and dose of ACS treatment in HDP and non‐HDP cases.

	< 48 h partial	< 48 h full	48 h–7 day	7 day–1 month	>1 month	Unknown	Total
HDP	590 (15.2)	621 (15.9)	1243 (31.9)	897 (23.0)	416 (10.7)	127 (3.3)	3894 (100)
Non‐HDP	2651 (13.6)	2202 (11.3)	4578 (23.4)	4600 (23.6)	4377 (22.4)	1115 (5.7)	19 523 (100)
Total	3241 (13.8)	2823 (12.1)	5821 (24.9)	5497 (23.5)	4793 (20.5)	1242 (5.3)	23 417 (100)

*Note*: Data are presented as *n* (%).

Abbreviations: ACS, antenatal corticosteroid; HDP, hypertensive disorders of pregnancy.

**FIGURE 3:**
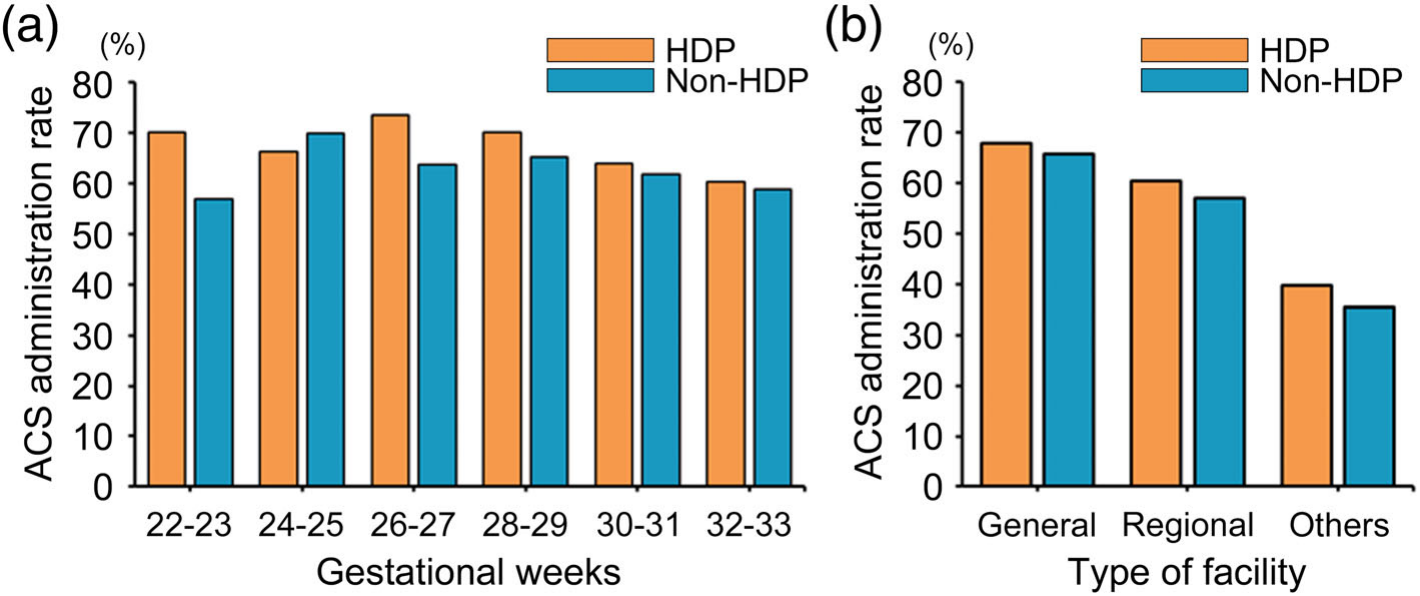
ACS administration rates stratified by gestational weeks and type of facility in patients with and without HDP between 2020 and 2022. (a) The ACS administration rate stratified by gestational age in patients with and without HDP and non‐HDP between 2020 and 2022. (b) ACS administration rates stratified by facility type (general perinatal medical centers, regional perinatal medical centers, and others) in patients with and without HDP and non‐HDP between 2020 and 2022. ACS, antenatal corticosteroid; HDP, hypertensive disorder of pregnancy.

**FIGURE 4:**
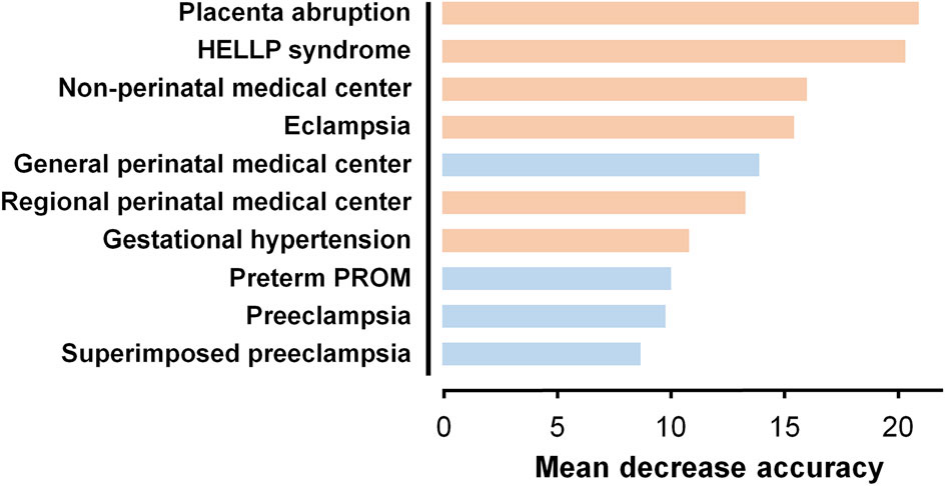
Feature importance of factors associated with non‐administration of ACS in HDP cases. The top 10 features based on the mean decrease in accuracy from the Random Forest analysis are listed. Features at the top indicate higher importance. The features highlighted in red indicate the promotion of non‐ACS treatment, whereas those highlighted in blue indicate suppression of non‐ACS treatment. Because the Random Forest algorithm cannot indicate the direction of feature importance, color coding was performed based on the crude OR, as shown in Table 4 to enhance interpretability. HELLP syndrome, hemolysis, elevated liver enzymes, and low platelet count syndrome; PROM, premature rupture of membranes.

In Analysis 3, a comparison of ACS administration rates between HDP and non‐HDP cases across gestational weeks at delivery and type of facility was conducted for 22 808 patients (4307 HDP and 18 501 non‐HDP patients) delivered before 34 weeks of gestation between 2020 and 2022. The ACS administration rates across gestational weeks are shown in Figure 3a. ACS administration rates differed according to the facility type (Figure 3b). For patients with HDP, the rates were 67.8% (1641/2419), 60.5% (1107/1830), and 39.7% (23/58) in general, regional, and non‐perinatal medical centers, respectively. For patients without HDP, the rates were 65.7% (7033/10709), 56.9% (4225/7419), and 35.4% (132/373) in general, regional, and non‐perinatal medical centers, respectively. Among the 4307 patients with HDP, ACS was not administered to 1,536 (35.7%) patients. The maternal and neonatal characteristics of the HDP patients with and without ACS treatment are detailed in Table 3. To identify the factors associated with non‐administration of ACS in patients with HDP, univariate and multivariate logistic regression analyses were performed (Table 4). Several factors were significantly associated with the non‐administration of ACS, including smoking during pregnancy (1.49 [1.00–2.21]), placental abruption (1.99 [1.47–2.69]), eclampsia (2.40 [1.08–5.32]), HELLP (hemolysis, elevated liver enzymes, and low platelet count) syndrome (1.46 [1.12–1.89]), gestational hypertension (1.42 [1.17–1.73], with preeclampsia as the reference group), regional perinatal medical centers (1.19 [1.02–1.39] with general perinatal medical centers as the reference group), and non‐perinatal medical centers (2.71 [1.48–4.99] with general perinatal medical centers as the reference group). Conversely, factors associated with decreased risk for non‐administration of ACS included fetal growth restriction (adjusted OR 0.68 [0.58–0.79]), threatened preterm labor (adjusted OR 0.71 [0.58–0.89]), and preterm premature rupture of membranes (adjusted OR 0.41 [0.27–0.63]). Figure 4 shows the feature importance of factors associated with the non‐administration of ACS in patients with HDP. Top‐ranking factors included emergency HDP‐related complications such as placental abruption, HELLP syndrome, and eclampsia. Non‐perinatal and regional medical centers and gestational hypertension were identified as important factors for the non‐administration of ACS.

**TABLE 3:** Maternal and neonatal characteristics in the HDP cases with and without ACS treatment.

	ACS	Non‐ACS	
Maternal characteristics	*n* = 2771	*n* = 1536	*p*‐value
Maternal age (years)	34.6 ± 5.2	34.5 ± 5.4	0.57
Primipara	1646/2757 (59.7)	875/1529 (57.2)	0.12
Pre‐pregnancy BMI > 25 (kg/m^2^)	613/2084 (29.4)	315/1034 (30.5)	0.55
BMI at delivery >25 (kg/m^2^)	1031/1810 (57.0)	548/915 (59.9)	0.14
Gestational week at delivery	30.7 ± 2.7	31.1 ± 2.6	<0.01
Cesarean section	2670/2770 (96.4)	1460/1535 (95.1)	0.04
NRFS (Level 4–5)	268/1401 (19.1)	141/702 (20.1)	0.60
Maternal transfer	1133/2771 (40.9)	699/1536 (45.5)	<0.01
ART	528/2765 (19.1)	247/1530 (16.1)	0.02
Smoking during pregnancy	64/2143 (3.0)	46/1087 (4.2)	0.07
Drinking during pregnancy	15/2056 (0.7)	9/1041 (0.9)	0.69
Gestational diabetes mellitus	197/2771 (7.1)	96/1536 (6.3)	0.28
Eclampsia	15/2771 (0.5)	22/1536 (1.4)	<0.01
Placenta abruption	118/2771 (4.3)	141/1536 (9.2)	<0.01
HELLP syndrome	211/2771 (7.6)	164/1536 (10.7)	<0.01
Fetal growth restriction	1177/2771 (42.5)	497/1536 (32.4)	<0.01
Threatened preterm labor	502/2771 (18.1)	206/1536 (13.4)	<0.01
Preterm PROM	152/2771 (5.5)	37/1536 (2.4)	<0.01
Placenta previa	40/2771 (1.4)	15/1536 (1.0)	0.19
Subtype of HDP			<0.01
Preeclampsia	1713/2728 (62.8)	906/1514 (59.8)	
Gestational hypertension	462/2728 (16.9)	336/1514 (22.2)	
Superimposed preeclampsia	420/2728 (15.4)	185/1514 (12.2)	
Chronic hypertension	133/2728 (4.9)	87/1514 (5.7)	
Multiple pregnancy	256/2767 (9.3)	98/1533 (6.4)	<0.01
Birth weight	1208 ± 448	1331 ± 467	<0.01
Small for gestational age	1724/2745 (62.8)	800/1526 (52.4)	<0.01
Type of facility			<0.01
General perinatal medical centers	1641/2771 (59.2)	778/1536 (50.7)	
Regional perinatal medical centers	1107/2771 (39.9)	723/1536 (47.1)	
Non‐perinatal medical centers	23/2771 (0.8)	35/1536 (2.3)	

*Note*: Data are presented as *n* (%) or mean ± standard deviation.

Abbreviations: ACS, antenatal corticosteroid; ART, assisted reproductive technology; BMI, body mass index; HELLP syndrome, hemolysis, elevated liver enzymes, and low platelet count syndrome; HDP, hypertensive disorders of pregnancy; NRFS, non‐reassuring fetal status; PROM, premature rupture of membranes.

**TABLE 4:** Factors associated with non‐administration of ACS in HDP cases.

	Crude OR (95% CI)	Adjusted OR (95% CI)
Maternal transfer	1.21 (1.07–1.37)	1.15 (0.98–1.34)
ART	0.82 (0.69–0.96)	0.93 (0.76–1.14)
Smoking during pregnancy	1.44 (0.98–2.11)	1.49 (1.00–2.21)
Eclampsia	2.67 (1.38–5.16)	2.40 (1.08–5.32)
Placenta abruption	2.27 (1.77–2.93)	1.99 (1.47–2.69)
HELLP syndrome	1.45 (1.17–1.80)	1.46 (1.12–1.89)
Fetal growth restriction	0.65 (0.57–0.74)	0.68 (0.58–0.79)
Threatened preterm labor	0.70 (0.59–0.84)	0.71 (0.58–0.89)
Preterm PROM	0.43 (0.30–0.61)	0.41 (0.27–0.63)
Subtype of HDP		
Preeclampsia	1.0 (reference)	1.0 (reference)
Gestational hypertension	1.38 (1.17–1.62)	1.42 (1.17–1.73)
Superimposed preeclampsia	0.83 (0.69–1.01)	0.89 (0.71–1.12)
Chronic hypertension	1.24 (0.93–1.64)	1.40 (0.99–1.98)
Multiple pregnancy	0.67 (0.53–0.85)	0.80 (0.60–1.07)
Type of facility		
General perinatal medical centers	1.0 (reference)	1.0 (reference)
Regional perinatal medical centers	1.38 (1.21–1.56)	1.19 (1.02–1.39)
Non‐perinatal medical centers	3.21 (1.88–5.47)	2.71 (1.48–4.99)

*Note*: Multivariable logistic regression analyses were performed using the forced entry method to identify factors associated with the non‐administration of ACS in patients with HDP. Variables with a *p*‐value <0.10 in the univariate logistic regression analysis were included in the multivariable logistic regression analysis. In the multivariate analysis, 3113 (72.3%) of 4307 cases were analyzed after excluding 1194 cases with missing values. The results were reported as crude and adjusted odds ratios (ORs) and 95% confidence intervals (CIs). The results for the HDP subtype were reported as OR and 95% CI, with preeclampsia as the reference group. The results of the type of facility are reported as OR and 95% CI, with general perinatal medical centers as the reference group.

Abbreviations: ACS, antenatal corticosteroid; ART, assisted reproductive technology; CI, confidence interval; HDP, hypertensive disorders of pregnancy; HELLP syndrome, hemolysis, elevated liver enzymes, OR, odds ratio; and low platelet count syndrome; PROM, premature rupture of membranes.

**FIGURE 5:**
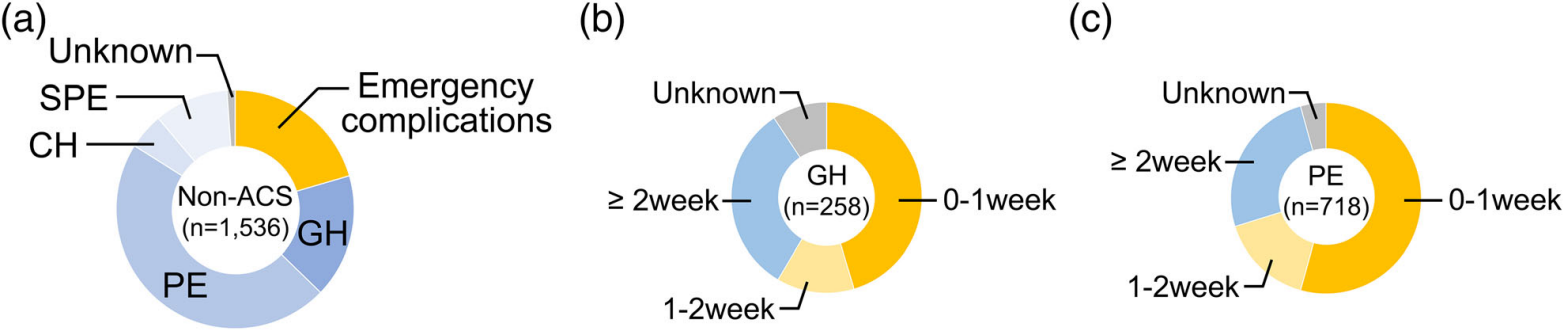
HDP subtypes and time interval from diagnosis to delivery in the non‐ACS‐treated cases. (a) Subtypes of HDP in non‐ACS‐treated cases, excluding emergency complications (eclampsia, HELLP syndrome, and placental abruption). (b) Time interval between diagnosis and delivery in patients with gestational hypertension. (c) Time interval between diagnosis and delivery in patients with preeclampsia. ACS, antenatal corticosteroid; CH, chronic hypertension; GH, gestational hypertension; HDP, hypertensive disorders of pregnancy; HELLP syndrome, hemolysis, elevated liver enzymes, and low platelet count syndrome; PE, preeclampsia; SPE, superimposed preeclampsia.

The proportion of emergency complications associated with HDP (eclampsia, HELLP syndrome, and placenta abruption) and of HDP subtypes among the non‐ACS‐administered cases (*n* = 1536) are displayed in Figure 5a. Emergency complications accounted for 20.5% of cases, whereas other HDP subtypes included gestational hypertension (16.8%) and preeclampsia (46.7%). In gestational hypertension patients without ACS treatment (*n* = 258), 45.3% (*n* = 117) delivered within 1 week of hospital admission, and 13.2% (*n* = 34) delivered within 2 weeks (Figure 5b). Similarly, in preeclampsia patients without ACS treatment (*n* = 718), 54.3% (*n* = 390) delivered within 1 week of hospital admission, and 15.8% (*n* = 114) delivered within 2 weeks (Figure 5c).

## DISCUSSION

This retrospective study investigated the current practice of ACS treatment in pregnancies complicated by HDP, using a nationwide, population‐based perinatal clinical database in Japan. Annual trends, timing of delivery, administration‐to‐birth intervals, disparities across gestational weeks and types of facilities, and clinical factors influencing non‐ACS administration were analyzed. The key findings of this study are^1^: ACS administration rates in patients with HDP have approximately doubled over the past decade^2^; approximately two‐thirds of HDP cases treated with ACS resulted in delivery before 34 weeks; however, only about 30% delivered within the optimal administration‐to‐birth interval of 48 hours to 7 days^3^; ACS administration rates for patients with HDP showed significant variations among the types of facilities^4^; the clinical factors associated with the inability to administer ACS to patients with HDP were identified. These findings provide important insights into the current practices of ACS treatment and highlight areas for potential improvement in the management of patients with HDP at high risk of preterm delivery.

The near doubling of ACS administration rates in patients with HDP and non‐HDP over the past decade is likely attributable to the introduction of insurance coverage of ACS treatment in 2009 and the dissemination of Japanese obstetric clinical practice guidelines among healthcare professionals. However, internationally, ACS administration rates in high‐income countries tend to be higher, with studies reporting rates exceeding 80%–90% in preterm pregnancies,^15^, ^16^, ^17^ highlighting the need for further efforts to improve ACS administration rates in Japan.

Although ACS administration rates for patients with HDP have consistently been higher than those among patients without HDP in most years, the difference remains relatively modest. The efficacy of ACS treatment is well‐established, regardless of HDP status, and the Japanese obstetric clinical practice guidelines are consistent with the international standards for ACS treatment.^7^, ^14^ ACS treatment is recommended in cases in which preterm delivery before 34 weeks is anticipated within 1 week. In this study, approximately 70% of HDP cases delivered before 34 weeks, while only about 7% delivered at 37 weeks or later. Compared to international data, the proportion of ACS‐treated cases that delivered before 34 weeks and at or beyond 37 weeks was 30–40% and approximately 40%, respectively (although these data are not limited to HDP cases),^18^, ^19^, ^20^ indicating that the delivery rate before 34 weeks in Japan is favorable and that guideline‐recommended practices have been effectively implemented in Japan.

However, only approximately 30% of patients with HDP achieved the optimal administration‐to‐birth interval of 48 h to 7 days following ACS administration, the period during which the treatment benefits are most pronounced. Additionally, this proportion of optimal timing of ACS administration was comparable to that reported in other countries that were not limited to HDP cases.^21^, ^22^ Although this proportion is notable, it highlights the inherent challenges in predicting the timing of preterm delivery and optimizing ACS administration. This is because HDP is a heterogeneous condition, with considerable variability in disease severity, onset, progression, and HDP‐associated emergency complications. These factors can influence clinical decisions regarding ACS administration and hinder the optimal administration timing. Recently, using risk scores and biomarkers, such as soluble fms‐like tyrosine kinase‐1/placental growth factor ratio, for predicting HDP deterioration has been reported.^23^, ^24^, ^25^, ^26^ Our study emphasizes the need to establish biomarkers that assist in predicting disease progression and determining the optimal delivery time for cases with HDP, as well as the importance of developing management strategies that integrate such prediction biomarkers into HDP care.

The marked variation in ACS administration rates among the different types of facilities may reflect disparities in clinical practice across Japan. Although the reasons for this remain unclear, physician awareness of ACS, level of training and education among healthcare providers, management strategies for HDP and preterm birth, and coordination of maternal transfer systems among primary, secondary, and tertiary care facilities may play a role. Further investigation is necessary to identify the factors contributing to these disparities. However, these differences should be interpreted with caution. Although approximately 20% of the cases included in this perinatal clinical database were delivered at non‐perinatal medical centers, only approximately 1% of preterm births delivered before 34 weeks were managed at these non‐perinatal medical centers. It is likely that a significant proportion of cases managed by non‐perinatal facilities, which do not typically handle preterm deliveries before 34 weeks, involved emergency situations requiring urgent delivery. This suggests that the differences observed may reflect the urgency of the cases rather than facility‐based disparities.

In cases of HDP, barriers to ACS administration include smoking during pregnancy, eclampsia, placental abruption, HELLP syndrome, gestational hypertension, regional perinatal medical centers, and non‐perinatal medical centers. Eclampsia, placental abruption, and HELLP syndrome often necessitate immediate delivery, leaving insufficient time for full administration of ACS. In such cases, the inability to administer ACS is understandable, as the priority is focused on life‐saving care for both the mother and fetus. However, smoking during pregnancy, which was identified as a significant factor in this study, is a modifiable behavior. Conversely, factors associated with increased ACS administration rates in patients with HDP include fetal growth restriction, threatened preterm labor, and preterm premature rupture of membranes. These conditions often involve planned interventions and provide a window for administering ACS.

The reasons for the lower administration rate in gestational hypertension among the HDP subtypes remain unclear; however, two possible explanations can be proposed. First, as gestational hypertension does not necessarily require inpatient management, determining the appropriate timing for hospitalization may be more difficult than in cases of preeclampsia or SPE, potentially leading to missed opportunities for timely ACS administration. Second, the absence of organ dysfunction, such as proteinuria and fetal growth restriction, may have made it more challenging to determine the optimal timing for ACS administration. Additionally, obstetrician‐gynecologists may have been unable to recognize the rapid progression of HDP, resulting in unexpected disease advancement, necessitating delivery before ACS could be administered.

In the analysis of 1536 non‐ACS‐treated HDP cases, approximately 20% involved obstetric emergency complications. However, considering that approximately half of the gestational hypertension and preeclampsia cases had an interval of more than 1 week from diagnosis to delivery, there is the potential to further increase the ACS administration rate in this timeframe. These results suggest the need to reconsider the timing of ACS administration to ensure that opportunities for ACS treatment are not missed in non‐emergency cases. This finding underscores the potential to increase ACS administration rates through strategies such as early transfer to perinatal medical centers, regular and careful monitoring of maternal and fetal conditions, and preparation for emergency situations. Moreover, raising clinicians' awareness about the importance of ACS is critical for reducing disparities in care.

This study used a comprehensive, population‐based perinatal database with a large sample size, enabling detailed subgroup analyses and identifying barriers to ACS administration. Furthermore, the focus on HDP cases provided useful insights into a high‐risk maternal and neonatal population, a topic that has been underexplored in previous research.

However, this study had several limitations. The database did not include certain factors that may influence ACS administration, such as clinical decision‐making processes of healthcare providers and detailed clinical information (e.g., HDP severity, blood test results, blood pressure, proteinuria, and the duration between hospitalization and delivery). Furthermore, the study did not include delivery data from primary care facilities, which may limit the generalizability of the findings. Nonetheless, given that deliveries before 34 weeks are predominantly managed in perinatal medical centers, this limitation likely had minimal impact on our conclusions. Finally, the reliability of data from the obstetric clinical database represents a significant limitation, as the accuracy of these records has not been formally validated. Although this study may have minimized the impact of outliers through statistical processing, further validation using other databases, such as the Diagnosis Procedure Combination database and the Neonatal Research Network Japan database, is necessary.

Three key steps should be considered to improve the rate and optimize ACS administration in Japan. First, it is essential to ensure that obstetrician‐gynecologists fully recognize the efficacy and importance of ACS. Second, promoting a clear understanding of the current status and challenges of ACS use in Japan—which was the primary objective of this study—is crucial. Third, efforts should be made to identify predictive biomarkers that can indicate the need for delivery within 1 week in pregnant women with HDP, thereby enabling the early identification of patients at risk of disease progression. These measures can improve the rate and timing of ACS administration.

In conclusion, our findings provide an overview of the current practice of ACS treatment for HDP in Japan and highlight significant progress in ACS administration rates for patients with HDP over the past decade. Addressing the underlying factors contributing to barriers to ACS treatment, including facility and clinical disparities, through early maternal transfer, careful monitoring, preparedness for obstetric emergencies, and increased awareness of the efficacy of ACS treatment could enhance the ACS administration rate and improve neonatal outcomes in HDP‐complicated pregnancies.

## AUTHOR CONTRIBUTIONS

**Takafumi Ushida:** Conceptualization; data curation; formal analysis; writing – original draft; methodology; investigation; funding acquisition; visualization; validation. **Kazuya Fuma:** Conceptualization; data curation; formal analysis; methodology; investigation; writing – review and editing. **Satoru Katsuki:** Conceptualization; formal analysis; methodology; investigation; writing – review and editing. **Sho Tano:** Conceptualization; formal analysis; methodology; investigation; writing – review and editing. **Seiko Matsuo:** Conceptualization; formal analysis; methodology; investigation; writing – review and editing. **Kenji Imai:** Conceptualization; formal analysis; methodology; investigation; writing – review and editing. **Hiroaki Kajiyama:** Conceptualization; formal analysis; methodology; investigation; supervision; writing – review and editing. **Tomomi Kotani:** Conceptualization; formal analysis; methodology; investigation; supervision; writing – review and editing.

## CONFLICT OF INTEREST STATEMENT

The authors declare no competing financial interests or personal relationships that could have influenced the conduct or reporting of this study. Hiroaki Kajiyama is the editor‐in‐chief of the Journal of Obstetrics and Gynecology Research and a co‐author of this article. Thus, he was excluded from editorial decision‐making related to the acceptance and publication of this article; rather, an associate editor independently handled all editorial decision‐making to minimize bias.

## ETHICS STATEMENT

This study was approved by the Ethics Committee of Nagoya University Hospital (approval no. 2024‐301; approval date: November 11, 2024).

## Supporting information


**Figure S1.** Observed distribution of ACS treatment rates in 2022.

## Data Availability

Data supporting the findings of this study are available from the Japan Society of Obstetrics and Gynecology. Restrictions apply to the availability of the data used under a license for the current study. Data are available with permission from the Japan Society of Obstetrics and Gynecology.
