# Supplementary figures and images for: Antenatal corticosteroid treatment for women with hypertensive disorders of pregnancy: A population‐based study in Japan
Source: J Obstet Gynaecol Res. 2025 Jul 2;51(7):e16364. doi: 10.1111/jog.16364 (PMC12223469; doi:10.1111/jog.16364)

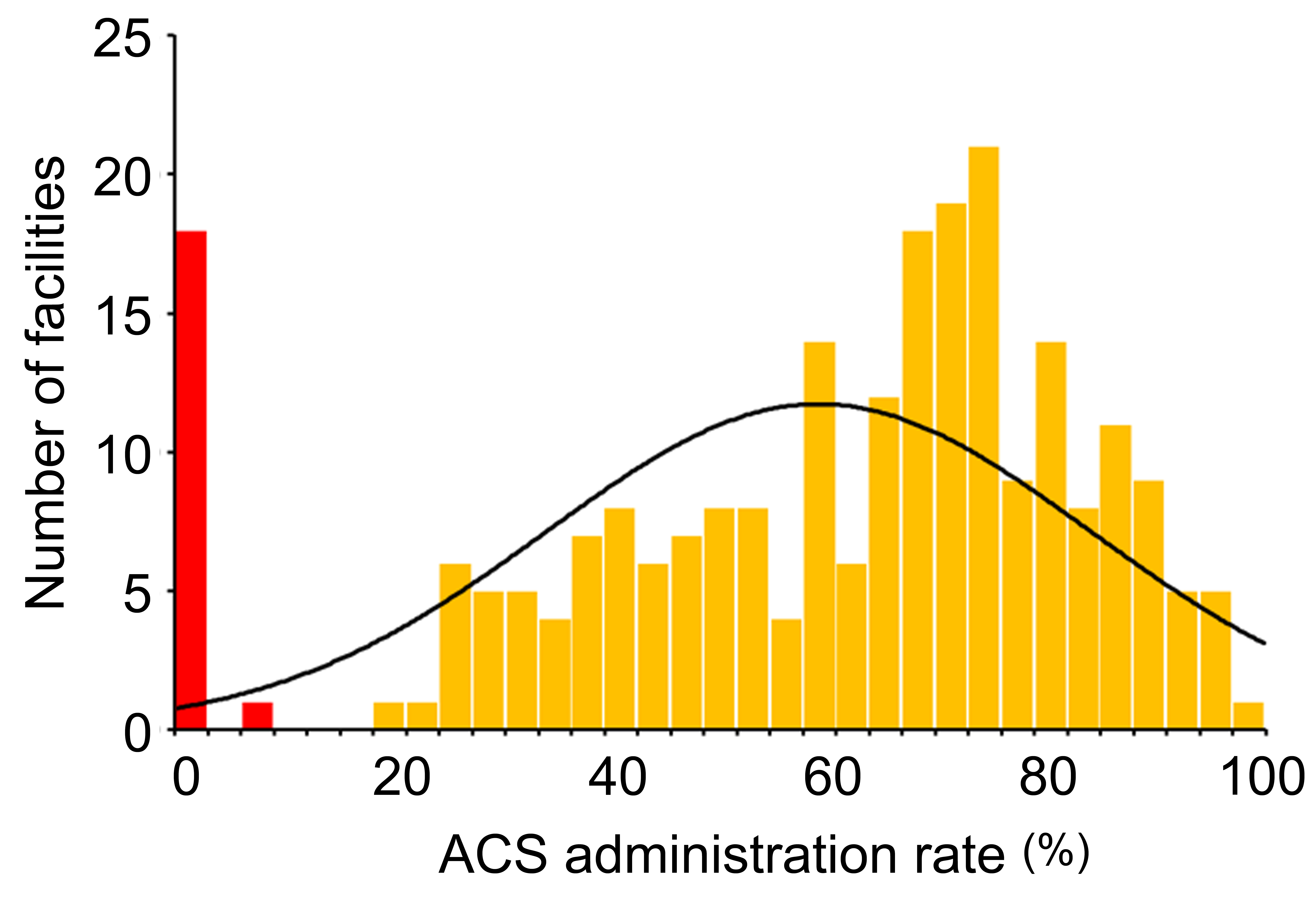

Supplement: Supplementary file 1 — Figure S1. Observed distribution of ACS treatment rates in 2022. [file JOG-51-0-s001.jpg]
